# *In utero* antidepressant exposure not associated with ADHD in the offspring: A case control sibling design

**DOI:** 10.3389/fphar.2022.1000018

**Published:** 2022-11-10

**Authors:** C. A. M. Hartwig, R. Robiyanto, S. de Vos, J. H. J. Bos, E. P. van Puijenbroek, E. Hak, C. C. M. Schuiling-Veninga

**Affiliations:** ^1^ PharmacoTherapy, Epidemiology and Economics, Groningen Research Institute of Pharmacy, University of Groningen, Groningen, Netherlands; ^2^ Program Studi Farmasi, Fakultas Kedokteran, Universitas Tanjungpura, Pontianak, Indonesia; ^3^ Pharmacovigilance Centre Lareb, ‘s-Hertogenbosch, Netherlands

**Keywords:** ADHD, antidepressants, case-control study, confounding by indication, *in utero* exposure, pregnancy, sibling design

## Abstract

Recent studies have reported an association between antidepressant (AD) use during pregnancy and the risk to develop attention-deficit/hyperactivity disorder (ADHD) in the offspring. However, the association might be confounded by risk factors in the pregnant parent. To control for unmeasured factors between pregnancies carried by the same parent, we set up a case-control sibling study using the University of Groningen prescription database IADB.nl. Children receiving medication for ADHD (cases) before the age of 16 years were matched to siblings not receiving such medication (controls). Exposure was defined as at least two prescriptions for any AD during pregnancy, i.e., the period of 39 weeks before the birth date of the offspring. Secondary analyses were performed to assess the effects of the degree of exposure (the amount of Defined Daily Doses) and the type of AD exposed to. Univariate and multivariate logistic regression was used to estimate odds ratios (ORs) with corresponding 95% confidence intervals (CI). In total, 2,833 children (1,304 cases and 1,529 controls) were included in the analysis. Exposure rate to ADs among cases and controls was 2.2% and 2.4%, respectively. After adjusting for the birth date of the child (as a proxy for the date of pregnancy), age of the pregnant parent at birth, use of psychostimulants, opioids, and antiepileptic drugs by the pregnant parent in the 15 months before birth of the child, an adjusted OR of 1.11 (95% CI 0.67–1.83) was found for the risk of ADHD in the offspring when exposed *in utero* to ADs. This indicates no increased risk of ADHD in offspring following *in utero* exposure to ADs. The secondary analyses revealed no statistically significant associations either. The present study provides further evidence that an association between *in utero* AD exposure and ADHD in offspring might not exist. This perceived association may be caused (at least partially) by confounding by indication. The extent to which depression in the pregnant parent could cause mental disorders such as ADHD in offspring, and the mechanisms involved, should be investigated in further studies.

## Introduction

The rate of depression during pregnancy is approximately 17% according to a meta-analysis on 15 studies from year 2000–2016, with rates ranging between 4.8% and 33.2% (Underwood et al., 2016). Untreated severe depression during pregnancy has been associated with increased incidence of adverse birth outcomes such as premature birth and low birth weight (Bonari et al., 2004; Meltzer-Brody, 2011). This association may be caused by unhealthy lifestyle and poor adherence to prenatal care by depressed parents, which could harm both parent and child. Therefore, it is important to treat perinatal depression by choosing safe treatment for the pregnant parent and the unborn child after careful consideration.

A descriptive drug utilization study on six European regions found that a weighted average of 2.5% of pregnancies in the period 2004–2010 were exposed to selective serotonin reuptake inhibitors (SSRIs) (Charlton et al., 2015). According to a Dutch study (Ververs et al., 2006), 2% of childbearing parents take antidepressants (ADs) during pregnancy. ADs are mainly prescribed for depression and anxiety (approximately 60% of prescriptions), but they may also be prescribed for other indications, e.g., obsessive–compulsive disorder or sleeping disorders (Gardarsdottir et al., 2007). Assessing the safety of antidepressant use during pregnancy is impeded by several challenges such as adjusting for confounding by indication, the severity of the underlying disease, and family history (Salas et al., 1999; Morales et al., 2018).

In recent years, a potential association between AD use during pregnancy and an increased risk of attention-deficit/hyperactivity disorder (ADHD) in offspring has been studied (Laugesen et al., 2013; Malm et al., 2016; Boukhris et al., 2017; Lupattelli et al., 2021). A meta-analysis on 7 studies found an adjusted risk ratio of 1.38 [95% confidence interval (CI) 1.13–1.69] for the risk of ADHD in offspring when comparing prenatal exposure to ADs to unexposed pregnancies (Morales et al., 2018). The popularization of the dopamine theory of ADHD (Levy, 1991; Swanson et al., 2007) led to an increased interest in evaluating potential associations between drugs and other environmental factors that may influence the dopaminergic system during fetal development and ADHD (Swanson et al., 2007; Figueroa, 2010). The suggestion of an association between *in utero* drug exposure and an increased risk of ADHD in offspring is disputable though, as the etiological mechanisms involved in this disorder are still largely unknown (Dmitrzak-Weglarz et al., 2021). Some studies find an association between AD exposure (specifically SSRI exposure) and ADHD, but when accounting for the psychiatric condition(s) of the childbearing parent, the association between SSRIs and ADHD often disappears (Malm et al., 2016), although this is not always the case (Boukhris et al., 2017). However, the latter study could not rule out confounding by severity of the indication entirely. The observed association also diminishes when comparing siblings from the same womb (Laugesen et al., 2013). Using a sibling comparison design will match familial factors such as genetics of the childbearing parent (Frisell et al., 2012). Since ADHD is highly heritable (Coghill and Banaschewski, 2009), it is likely that genetic risk factors are the main contributors to the disorder’s etiology, while non-inherited factors play a role as well (Thapar et al., 2012; Rahman et al., 2021; Kraegeloh-Mann, 2022). This makes a sibling study design suitable for investigating confounding by indication.

The current study aims to investigate the potential association between *in utero* AD exposure and risk of ADHD in offspring using data from the Dutch IADB.nl prescription database, while adjusting for confounding by indication by employing a sibling design. Secondarily, this study aims to investigate whether the degree of exposure and the specific type of AD exposed to are effect modifiers.

## Methods

### Setting and study design

A case-control study was conducted using data from a pregnancy database as part of the University of Groningen prescription database IADB.nl. The overarching database contains prescription data from 1994 until 2020 from approximately 120 community pharmacies, covering more than 1.1 million patients. In the pregnancy database used in this study, prescription data from 65,251 infants and their childbearing parents who were born between 1995 and 2020 were included. An address code is used to connect the childbearing parent and their child (Schirm et al., 2004). The childbearing parent is identified as a subject registered as female between the ages of 15 and 50 who resides at the same address as the child. This method of matching children and parents has been validated and demonstrated a 99.4% accuracy for matching childbearing parent to child correctly (Schirm et al., 2004). Throughout the database period, each person is tracked individually, and prescription records include data on the date of dispensing, the amount dispensed, the dose regimen, the number of days the prescription is valid, the prescribing physician, and the Anatomical Therapeutic Chemical (ATC) code (Sediq et al., 2018). Date of birth and sex are known, and each patient has their own unique anonymous identifying number. The database has been extensively used for research as it has been determined that the database’s population’s age, sex, and prescription rates are representative of the Netherlands as a whole (Sediq et al., 2018). Registration in the database is independent of health care insurance. With the exception of over-the-counter (OTC) drugs and drugs given during hospitalization, each patient’s medication records are nearly complete in the Netherlands because of its high patient-pharmacy commitment (Visser et al., 2013; Sediq et al., 2018).

### Study population

The study population was restricted to singleton pregnancies where the offspring could be followed from birth until at least 4 years. Because of this, all births included in the study took place between 1995 and 2016. The childbearing parent had to be in the database for at least 6 months before the pregnancy, which corresponds to 15 months before the birth of the child. The pregnancy period was defined as 273 days (or 39 weeks) before the birth date of the child.

### Case and control definition

Drug prescription data was used as a proxy for ADHD in offspring, as diagnostic information was not available. Guidelines for the treatment of ADHD recommend to start with behavioral therapy first and supplement with pharmacotherapy, beginning with methylphenidate (MPH) (Wolraich et al., 2011; Stijntjes et al., 2014). When response is inadequate, (lis-)dextroamphetamine and atomoxetine are prescribed for ADHD as alternatives. The majority of children with ADHD receiving medications, receive MPH: 86.8% in 2015 (Sluiter et al., 2020). In the Netherlands, the first prescription for MPH is usually received by children between 4 and 9 years old (Sluiter et al., 2020). In 75% of children receiving MPH, the first prescription was received when the child was 13 years old or younger. Therefore, a case of ADHD was defined as receiving at least two consecutive prescriptions for ADHD medication (i.e., MPH, dextroamphetamine, or atomoxetine) before the age of 16, with “consecutive” meaning the second prescription being received within 6 months. A control is defined as a sibling of a case, being born from the same womb, with no prescriptions for MPH, dextroamphetamine, or atomoxetine during follow-up until the 16th birthday. Cases were only included if they had at least one control sibling.

### Exposure definition

Exposure was defined as at least two prescriptions for any AD (ATC starting with: N06A) during pregnancy, with specific interest in SSRIs and tricyclic antidepressants (TCAs). Non-exposure was split into former users and never users. Former users were defined as childbearing parents who receive at least one prescription for any AD in the 6 months preceding pregnancy, but not during pregnancy. Never users are childbearing parents with no AD prescription anywhere during the 15 months before birth.

### Covariates

Among the covariates considered for confounding adjustment were sex of the offspring and age of the childbearing parent at delivery. Additionally, the current study takes into account whether other drugs than ADs were used by the childbearing parent in the 15 months before birth Specifically: psychostimulants (ATC: N06B), antipsychotics (ATC: N05A), benzodiazepine derivatives (ATC: N05BA; N05CD), opioids (ATC: N02A), antiepileptics (ATC: N03A), acid-suppressive drugs (ATC: A02B), drugs for obstructive airway diseases (ATC: R03), and ADHD drugs, i.e., MPH (ATC: N06BA04), dextroamphetamine (ATC: N06BA02) and atomoxetine (ATC: N06BA09). The moment in time the pregnancy took place was also taken into account by using the birth date of the child, as it is relevant for the changing prescription trends over time (Bachmann et al., 2017; Sluiter et al., 2020). In the childbearing parent and the offspring, use of respiratory drugs (ATC: R03) was taken into account as well. The reason for this is the apparent association between ADHD and atopic diseases, including asthma. A 2016 study using the IADB.nl prescription database found an increased odds of receiving treatment for ADHD in children with atopic diseases compared to children without atopic diseases (van der Schans et al., 2016). Aside from this, parents receiving treatment for asthma were more likely to have children with ADHD than parents who did not receive such treatment [adjusted OR (aOR) = 1.2, 95% CI 1.1–1.3], regardless of which parent received treatment and when this treatment occurred (van der Schans et al., 2016).

### Data analysis

**FIGURE 1:**
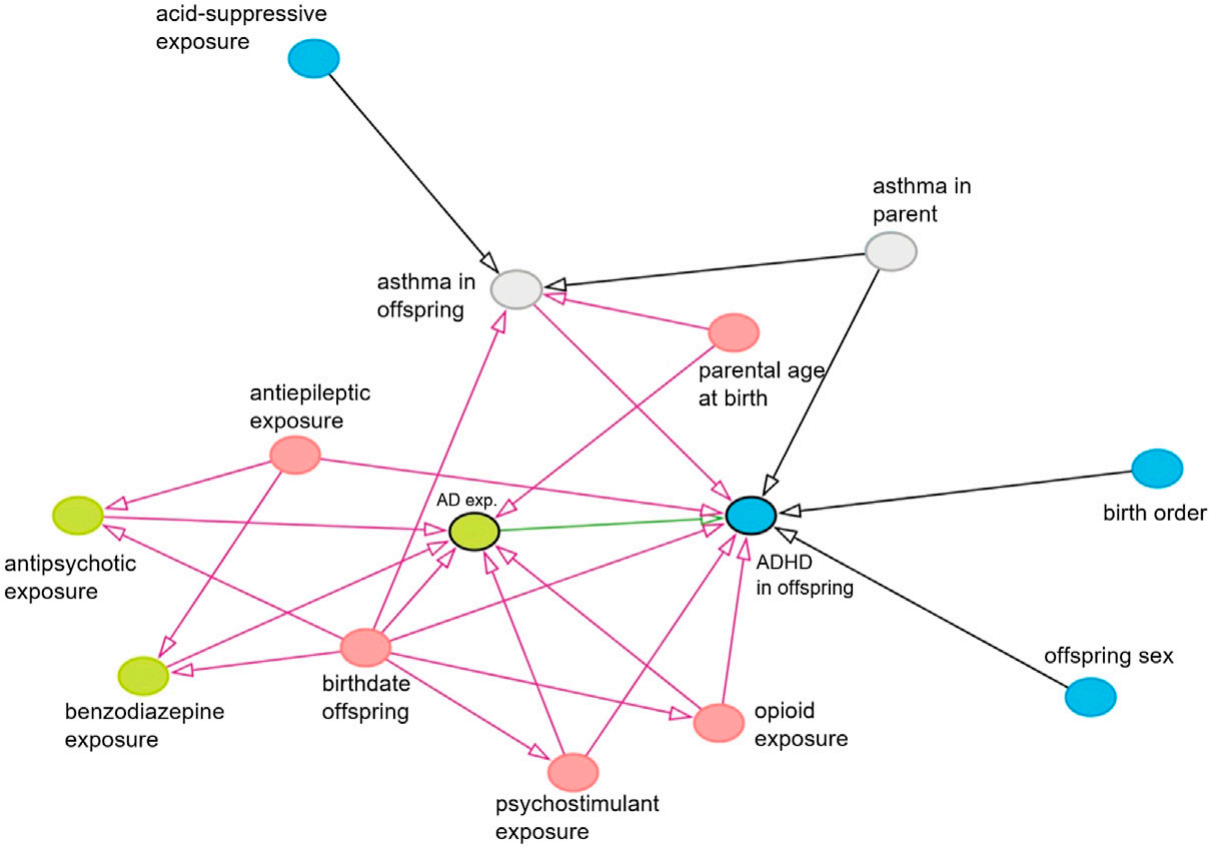
Causal diagram showing causal associations between all variables which have an association with the exposure and/or with the outcome. Only observed variables or unobserved mediators are shown. Exposure is shown in green with a black border, the effect of interest is shown as a green arrow, the outcome is shown in blue with a black border, and potential confounders and their biasing paths are shown in pink. Green and blue nodes are ancestors of exposure and outcome, respectively. Gray nodes are unobserved variables.

**FIGURE 2:**
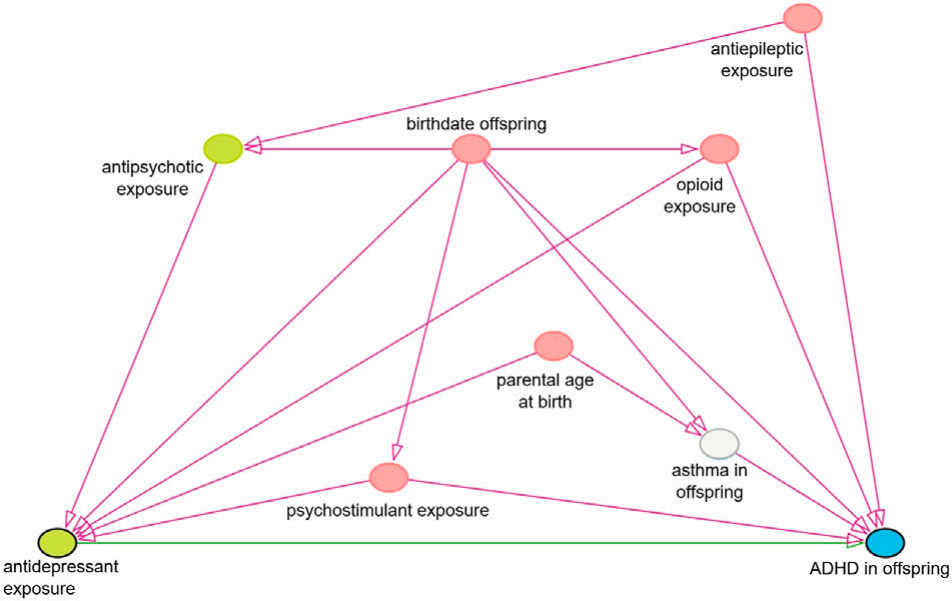
Simplified causal diagram showing causal associations between relevant variables. This diagram represents the associations relevant for the chosen minimal sufficient adjustment set for estimating the effect of *in utero* antidepressant exposure on the risk of ADHD in offspring. This adjustment set was chosen as it was the smallest out of four sets and consists of the five pink nodes in this diagram.

For the primary analysis, crude odds ratios (ORs) with corresponding 95% confidence intervals (95% CI) were determined by applying a logistic regression model using R. All unexposed pregnancies were used as reference, regardless of former user status. ORs were adjusted for confounders in multivariate logistic regression models. Confounders were determined to be the birth date of the child (as proxy for the date of pregnancy), age of the childbearing parent at birth and use of psychostimulants, opioids, and antiepileptic drugs by the childbearing parent in the 15 months before birth. The covariates for inclusion in the models were selected based on literature and on causal diagrams built using DAGitty (Figures 1, 2) (Shahar and Shahar, 2012; Williamson et al., 2014; Textor et al., 2016; VanderWeele, 2019). Some factors for which data was collected were not included in the models, since they were not part of the minimal sufficient adjustment set for estimating the direct effect of *in utero* AD exposure on ADHD in offspring. This data can be found in an extended version of Table 1 in the supplementary materials.

**TABLE 1:** Baseline characteristics of 2,833 singleton pregnancies in the IADB.nl prescription database, with comparison between cases and controls.

	Cases	Controls	Total
	(*n* = 1,304)	(*n* = 1,529)	(*n* = 2,833)
Offspring			
Age at first ADHD prescription (years)			
Mean (SD)	8.74 (2.73)	N/A	N/A
Median [min, max]	8 [2, 15]		
0–5	107 (8.2%)		
6–10	878 (67.3%)		
11–15	319 (24.5%)		
Follow-up time (years)			
Mean (SD)	15.3 (3.99)	13.2 (5.59)	14.1 (5.03)
Median [min, max]	15.2 [5.32, 25.6]	12.7 [4.00, 25.5]	14.4 [4.00, 25.6]
Sex			
Male	989 (75.8%)	725 (47.4%)	1,714 (60.5%)
Female	315 (24.2%)	804 (52.6%)	1,119 (39.5%)
Other/unknown	0 (0.0%)	0 (0.0%)	0 (0.0%)
Birth order			
Firstborn	722 (55.4%)	502 (32.8%)	1,224 (43.2%)
Later birth	582 (44.6%)	1,027 (67.2%)	1,609 (56.8%)
Concomitant drug use			
R03 drugs	575 (44.1%)	646 (42.2%)	1,221 (43.1%)
Beta 2 agonists	516 (39.6%)	580 (37.9%)	1,096 (38.7%)
Glucocorticoids	339 (26.0%)	368 (24.1%)	707 (25.0%)
Parent			
Age at delivery (years)			
Mean (SD)	28.6 (4.47)	29.5 (4.48)	29.1 (4.50)
Median [min, max]	29 [16, 48]	29 [17, 44]	29 [16, 48]
*In utero* AD exposure			
Exposed	29 (2.2%)	36 (2.4%)	65 (2.3%)
Unexposed	1,275 (97.8%)	1,493 (97.6%)	2,768 (97.7%)
Degree of exposure (nDDDs)			
Mean (SD)	198 (155)	232 (155)	217 (155)
Median [min, max]	210 [5.99, 675]	218 [15.0, 540]	210 [5.99, 675]
Antidepressant type^a^	** **	** **	** **
No AD	1,261 (96.7%)	1,486 (97.2%)	2,747 (97.0%)
TCA	12 (0.9%)	5 (0.3%)	17 (0.6%)
SSRI	27 (2.1%)	32 (2.1%)	59 (2.1%)
Other	4 (0.3%)	7 (0.5%)	11 (0.4%)
Time period of exposure^b^			
6 months before pregnancy	72 (34)	79 (45)	151 (79)
T1	37 (2)	33 (2)	70 (4)
T2 and/or T3	27 (1)	35 (5)	62 (6)
Concomitant drug use during the 15 months before delivery			
Psychostimulants	6 (0.5%)	6 (0.4%)	12 (0.4%)
Antipsychotics	7 (0.5%)	11 (0.7%)	18 (0.6%)
Benzodiazepines	93 (7.1%)	99 (6.5%)	192 (6.8%)
Opioids	24 (1.8%)	44 (2.9%)	68 (2.4%)
Antiepileptics	7 (0.5%)	3 (0.2%)	10 (0.4%)
Acid-suppressive drugs (ASDs)	61 (4.7%)	103 (6.7%)	164 (5.8%)

Abbreviations: N/A, not applicable; nDDDs, number of defined daily doses; AD, antidepressant; SSRI, selective serotonin reuptake inhibitor; TCA, tricyclic antidepressant; T1/2/3, trimester 1/2/3; ASDs, acid-suppressive drugs.

^a^ One control pregnancy switched from an AD, in the “other” group to an SSRI, during pregnancy. This pregnancy is counted in both groups.

^b^ The first number is not exclusive, e.g., a pregnancy exposed both during trimester 1 and before pregnancy is counted in both rows. The number in brackets is the exclusive exposure, that is to say the amount of pregnancies exposed exclusively during this period.

Two secondary analyses were performed. 1) The role of the degree of exposure was investigated by using the amount of Defined Daily Doses (a continuous variable) as the determinant instead of the binary variable of exposure status. 2) The effect of each AD type was determined by stratification, effectively leading to three separate analyses, one for each type of AD, being TCAs, SSRIs, and other ADs. For example, for the TCA-specific analysis, a subset of the sample data was used where SSRI-exposed pregnancies and pregnancies exposed to other ADs were removed. Note that these removed pregnancies were not classified as unexposed pregnancies. Additionally, an exploratory analysis was performed to investigate sex of the offspring as an effect modifier. Sex of the offspring was added to the adjusted model as well as an interaction term between sex and exposure.

## Results

From the IADB.nl prescription database, 1,304 cases of ADHD were identified and matched to 1,529 sibling controls without ADHD. The average age at which children with ADHD got their first prescription for ADHD medication was 8.74 years old (SD 2.73), with 1,294 (99.2%) cases starting with MPH and the rest starting with dextroamphetamine (Table 1). In total, 65 (2.3%) pregnancies were exposed to AD use: 29 (2.2%) in cases, 36 (2.4%) in controls. Over 75% of cases were male, while 47.4% of controls were male. 55.4% of children with ADHD were firstborn, while 32.8% of children without ADHD were firstborn. SSRIs were the most prevalent AD used during pregnancy (*n* = 59), followed by TCAs (*n* = 17), and other ADs (*n* = 11). 44.1% of children in the sample have used respiratory drugs. There was more use of acid-suppressive drugs during pregnancy in the control group when compared to the cases (*p* < 0.05).

In the main analysis of the association between AD exposure during pregnancy and ADHD in the offspring, a crude OR of 0.94 (95% CI 0.57–1.54) was determined (Table 2). The multivariate logistic regression model yielded an adjusted OR of 1.11 (95% CI 0.67–1.83). When taking the degree of exposure (a continuous variable) as the determinant in the model, an aOR of 1.000 (0.998–1.002) was found. When stratifying the type of AD that the pregnancy was exposed to, no statistical increases or decreases in the odds of ADHD in offspring following *in utero* exposure to ADs were found. For the exploratory analysis on sex of the offspring as an effect modifier, it was found that the interaction term was not significant: aOR = 0.76 (95% CI 0.24–2.24) with male sex as the reference.

**TABLE 2:** Crude and adjusted odds ratios for the development of ADHD in offspring after exposure to antidepressants during pregnancy.

	Crude OR (95% CI)	Adjusted OR (95% CI)^a^
Antidepressant (AD) exposure		
Any AD exposure (ATC N06A)	0.94 (0.57–1.54)	1.11 (0.67–1.83)
Degree of exposure		
nDDDs exposed (total)	0.999 (0.997–1.001)	1.000 (0.998–1.002)
AD type^b^		
TCA (*n* = 2,713)	2.36 (0.74–8.85)	1.43 (0.75–9.26)
SSRI (*n* = 2,778)	0.81 (0.44–1.47)	0.95 (0.50–1.74)
Other (*n* = 2,702)	0.71 (0.14–2.88)	1.06 (0.21–4.39)
Other		
Former user analysis (*n* = 50)	1.00 (0.27–3.74)	0.88 (0.19–3.92)

Abbreviations: OR, odds ratio; CI, confidence interval.

^a^ Adjusted for birth date of the child (as proxy for the date of pregnancy), age of the parent at birth, use of psychostimulants, opioids, and use of antiepileptic drugs in the 15 months before delivery.

^b^ Exposure to specific AD types was analyzed separately for each type. Each analysis used a different subset of the sample population, hence the varying sample sizes for each separate analysis.

## Discussion

The results of this study suggest the absence of an association between *in utero* exposure to ADs and the risk of developing ADHD in the offspring. The analyses on degree of exposure and the type of AD did not lead to any statistically significant differences either. The results of this study may imply that the previously observed association between prenatal AD use and increased incidence of ADHD in offspring (Malm et al., 2016; Boukhris et al., 2017) is actually caused not by the AD exposure itself, but by a confounder which is inherent to the childbearing parent of both siblings.

This study aimed to investigate the role of confounding by indication. In this case, the relevant confounding variable could be the pregnant parent’s depressive disorder or anxiety, which was the indication for them to start the use of ADs. It is likely that a parent’s general susceptibility to psychiatric disorders is a characteristic which could be easily passed on to their offspring genetically (Taylor et al., 2019; Cao et al., 2022), meaning that the children of parents with a psychiatric disorder would be more likely to develop psychiatric disorders themselves, regardless of the drug use of the parent. However, the indication might lead to an increased risk of psychiatric disorders in the offspring, not only because of a genetic predisposition, but also because of a higher discovery rate when compared to offspring of parents without psychiatric disorders (Figueroa, 2010). Psychiatric disorders in children are discovered more often when the parents also suffer from psychiatric disorders, due to an increased awareness of the existence of such disorders. This phenomenon is difficult to separate from confounding by indication and therefore, confounding by indication might be overestimated. In short, the results of the current study suggest that any observed association between AD use and ADHD in offspring might be explained (at least in part) by confounding by indication.

These results are in line with previous studies on the same association. A previous cohort study (Laugesen et al., 2013) found an aOR of 0.7 (95% CI 0.4–1.4) for the association between AD exposure between 30 days before pregnancy until birth and ADHD in offspring (based on diagnosis and/or prescriptions) when comparing siblings from the same womb. A more recent cohort study (Lupattelli et al., 2021) investigated the association between SSRI/SNRI exposure during pregnancy and risk of ADHD in offspring and calculated weighted hazard ratios (wHRs). When comparing hazard rates in exposed pregnancies to unexposed pregnancies, a wHR of 1.07 (95% CI 0.76–1.51) was found. When compared to former users of ADs who also reported no symptoms of depression or anxiety during pregnancy, wHR was 1.50 (95% CI 0.77–3.07). The authors concluded that prenatal SSRI/SNRI exposure is unlikely to considerably increase the risk of child ADHD beyond that posed by depression/anxiety in the pregnant parent. Such a comparison of former users would have a sample size of 50 pregnancies when using our data, which does not yield enough power to effectively investigate the association with this approach. Moreover, no data on symptoms experienced by pregnant people was available.

Our study adds to this previous evidence for the absence of an association in a different way than has been done before. Using causal diagrams is a better method for covariate selection than stepwise and univariate selection methods (Sun et al., 1996; Williamson et al., 2014; Smith, 2018). Nonetheless, residual confounding might still have occurred (Shahar and Shahar, 2012). A similar concern regarding residual confounding should be addressed with regard to using a sibling design. In general, the most straightforward way to account for unmeasured factors between pregnancies, is by comparing offspring of the same childbearing parent (Frisell et al., 2012). This makes matched sibling analyses suitable for investigating epidemiological research questions surrounding reproductive toxicology, e.g., how drug use during pregnancy affects the offspring.

Although matched sibling studies seem to be excellent for taking into account certain factors, there is still a risk of biases (Frisell et al., 2012). Confounding may still occur due to factors not completely shared by siblings. Furthermore, confounding bias may even be increased in sibling studies. This is caused by the fact that the siblings are selected in such a way that one has the outcome and the other does not. This selection suggests that these two siblings will differ more than two people randomly selected from the population who have the same outcome status, at least in terms of nonshared causes for the outcome. If one nonshared confounder causes the exposure and also causes the outcome or influences the probability of the outcome, the risk of (nonshared) confounding bias is increased in this sibling analysis (Frisell et al., 2012). It is therefore wise to assume that residual confounding is likely to occur and that characteristics shared by siblings might not be shared completely.

The population used in this study is from the IADB.nl prescription database, and is representative of the Netherlands as a whole (Visser et al., 2013; Sediq et al., 2018). A limitation of using prescription data is that prescriptions for ADHD medication do not equal diagnoses for ADHD. There is a possibility that only the more severe cases of ADHD are identified by using prescriptions, as not all children with ADHD receive psychostimulant therapy. In 2012, 48% of Dutch children with ADHD received psychostimulant therapy (Prins and van Dijk, 2015). Aside from this, MPH is not only prescribed for ADHD but also for narcolepsy and other indications (Sluiter et al., 2020). This could hamper interpretation of the results. Still, the majority of prescriptions for MPH to children are for ADHD and related symptoms (Donker et al., 2005) and prevalence of narcolepsy in children is low (Wijnans et al., 2013). The multidisciplinary guideline also recommends MPH as the initial treatment, with alternative medications solely as second-line options (Stijntjes et al., 2014). Dextroamphetamine and atomoxetine are only approved for treatment of ADHD according to their registration in the Netherlands. Another limitation is that it is unknown whether or not prescribed medication was actually taken when exclusively using prescription data. This issue was addressed by defining exposure as receiving two consecutive prescriptions for ADs. Furthermore, the indication behind the AD prescriptions is not known, nor is the status (presence/absence of active depressive episodes) or severity of depression during pregnancy. Similarly, no data on socioeconomical or lifestyle characteristics of the patients was available through the IADB.nl prescription database. This may influence the interpretation of the results. However, by using a sibling design the authors aimed to minimize the risk of confounding by indication, regardless of which indication that is. By using siblings, it is expected that the indication for the childbearing parent to use ADs is the same and therefore equally distributed among cases and controls. It should be noted that the genetic risk factors for psychiatric disorders in the parent are also controlled for by the sibling comparison design and genetic risk factors of ADHD are considered the main contributors to its etiology (Thapar et al., 2012; Rahman et al., 2021; Kraegeloh-Mann, 2022). Another limitation of the study is the unequal follow-up time for cases and controls, which may influence the comparison between siblings. Additionally, the date of conception of each pregnancy was not known and so the period of pregnancy was defined as 39 weeks before the birth date of the child for each pregnancy, 13 weeks per trimester. This may have led to misclassification of exposure, and made defining the boundaries of the trimesters less reliable, as some pregnancies are delivered prematurely and some are delivered after week 39. However, it is assumed that any misclassifications that could have been caused by this are random throughout the sample, regardless of ADHD status. We suggest for future investigations to stratify by trimester of exposure and look at pregnancies exclusively exposed during certain trimesters and not others. With the current data, an analysis pertaining to the trimester of exposure would not yield sufficient power, due to the low amount of pregnancies exposed during specific periods. For example, only 6 pregnancies were exposed to ADs exclusively during the first trimester (see Table 1).

Some characteristics of the population sample stand out. First of all, the lowest age of first prescription for ADHD medication was 2 years old (Table 1). In fact, 107 (8.2%) children with ADHD prescriptions received their first prescription before the age of six, which is the recommended minimum age to start with pharmacotherapy according to Dutch guidelines (Stijntjes et al., 2014). Thus, prescribing ADHD medication before the age of 6 is off-label. Comparable prevalence of off-label prescribing has been observed in a UK study of ADHD prescription trends, though (Beau-Lejdstrom et al., 2016), and it is recommended by Dutch guidelines to consider MPH treatment in children younger than 6 years old in severe cases if alternative treatment options are insufficient (Akwa GGZ, 2019).

Secondly, 75.8% of all ADHD cases in the sample were male, while the ratio male to female was closer to 1:1 in the group of controls. This skewed distribution is in line with previous studies on ADHD medication use in children (Reich et al., 2006; Derks et al., 2007; Rucklidge, 2010). In a Dutch twin study (Derks et al., 2007), it was found that only 6% of girls with ADHD received medication and 8% received counseling for ADHD, while this was 47% and 38% respectively in boys. Since the current study used prescriptions for medication to classify cases and controls, there is a possibility that many females with ADHD were misclassified as controls. It is impossible to say to what extent this misclassification could have been avoided by using official ADHD diagnoses, as it is also found that girls with ADHD are largely underdiagnosed when compared to boys with ADHD (Rucklidge, 2010). As sex of the offspring was deemed not to be a potential confounder or an effect modifier in the association between AD exposure during pregnancy and ADHD in offspring, the uneven distribution of sexes is assumed not to be a problematic factor in this study and might even demonstrate the representativeness of the database, precisely because of the aforementioned biases observed in Netherlands.

Finally, cases were more likely to be firstborn (55.4%) than controls (32.8%). According to prior studies, being the firstborn may be a risk factor for developing ADHD (Marín et al., 2014; Reimelt et al., 2021). However, diagnostic biases in firstborn children are more likely to blame for this observed birth-order influence (Reimelt et al., 2021). Furthermore, the current study does not have a balanced sample in terms of the case/control ratio. The uneven distribution of births could explain the larger proportion of later births among controls. When there are three or more siblings, only one can be the firstborn, whereas multiple siblings can be born later. The birth order was not included in the models, but the relevance of birth order on the risk of developing ADHD should be investigated further.

The present study provides further evidence that an association between *in utero* AD exposure and ADHD in offspring might not exist, but that this perceived association may be caused (at least partially) by confounding by indication. The extent to which depression in the pregnant parent could cause mental disorders such as ADHD in offspring and the mechanisms involved should be investigated in further studies, preferably using diagnostic data on both depression in the pregnant parent as well as on ADHD in the offspring.

## Data Availability

The data analyzed in this study is subject to the following licenses/restrictions: Data is stored in a secured surrounding (IADB.nl). Requests to access these datasets should be directed to info@iadb.nl.
